# Treatment of dogs with Bravecto^®^ (fluralaner) reduces mosquito survival and fecundity

**DOI:** 10.1186/s13071-023-05682-8

**Published:** 2023-04-28

**Authors:** Christopher Charles Evans, Dorothy Normile, Sheryl Gamble, Frank Guerino, Michael T. Dzimianski, Andrew Riddell Moorhead

**Affiliations:** 1 Department of Infectious Diseases, College of Veterinary Medicine, University of Georgia, Athens, GA USA; 2 Merck Animal Health, Madison, NJ USA

**Keywords:** Bravecto^®^ chewable tablets, Fluralaner, Dog, Mosquito, *Aedes aegypti*, Efficacy

## Abstract

**Background:**

Mosquitoes serve as the vector of canine heartworm (*Dirofilaria immitis*), which represents a significant and persistent threat to canine health. A reduction in the longevity and/or reproductive success of mosquitoes that take a blood meal from fluralaner-treated dogs may consequently reduce the local transmission of heartworm and prevent new infections. A novel secondary effect of an oral formulation of the ectoparasiticide fluralaner (Bravecto^®^) against a laboratory strain of the mosquito *Aedes aegypti*, a potential major vector of canine heartworm, was investigated in this study.

**Methods:**

Six dogs were administered a single dose of fluralaner orally in the form of Bravecto^®^ Chews (at the labeled fluralaner dose of 25 mg/kg body weight), while six control dogs received no treatment. Mosquitoes were fed on blood that was collected from each dog prior to treatment and weekly for 15 weeks post-treatment to assess the continued effects of fluralaner as its serum level decreased. Mosquito fitness was assessed by three parameters: rate of successful blood-feeding, survival, and egg laying.

**Results:**

Successful blood-feeding rate was similar between control and treatment groups. In the fluralaner treatment, mosquito survival was significantly reduced within the first 24 h after blood-feeding, for the first 12 weeks post-treatment of the dogs (efficacy range = 33.2–73.3%). Survival of mosquitoes up until a potentially heartworm-infective timepoint (14 days post-blood-feeding) was significantly reduced in the fluralaner-treated group at several timepoints (1, 2, 5, 11, 12, 13, 14, and 15 weeks post-treatment; efficacy range = 49.4–91.4%), but was less consistently reduced at the other timepoints. Egg laying by mosquitoes was almost completely suppressed for the first 13 weeks following treatment of the dogs with fluralaner (treatment efficacy ≥ 99.8%).

**Conclusions:**

Mosquitoes fed blood from fluralaner-treated dogs experienced a significant reduction in survival and fecundity. These findings support the potential for a reduction in heartworm transmission directly by lethal effects on the vector and indirectly through a reduction of the local vector population when mosquitoes are exposed to animals treated with fluralaner.

**Graphical Abstract:**

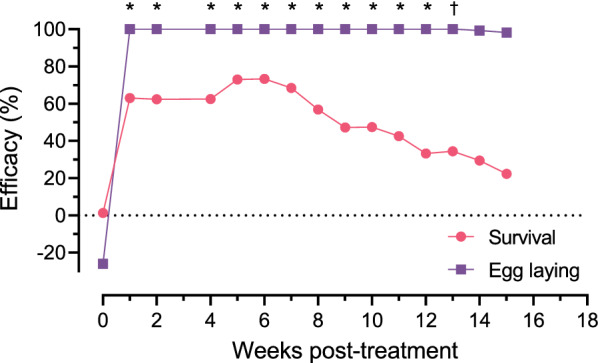

**Supplementary Information:**

The online version contains supplementary material available at 10.1186/s13071-023-05682-8.

## Background

While mosquitoes serve as vectors for several viral and bacterial pathogens of dogs, canine heartworm (*Dirofilaria immitis*) stands out as one of the most significant infectious diseases in companion animal health that is transmitted by mosquitoes. This filarial parasite has a broad distribution from tropical to temperate regions around the world and, due to its zoonotic potential, is also of concern for human health [1–3]. Furthermore, the expansion of its vector range and the increasing mobility of pets through travel and migration appear to be responsible for the introduction of heartworm to areas where it was once rare or absent [2, 4–6]. Several species of mosquito serve as potential vectors of heartworm, including *Aedes aegypti*, *Aedes albopictus*, and *Culex quinquefasciatus* [1]. In the United States, a national heartworm prevalence of 1.3% has been reported, with regional rates reaching 14.6–48.8% [7, 8]. Transmission is most intense during the warmer months, when mosquitoes are most active; the southern United States experiences especially high levels of transmission due to its climate, with prevalence rates increasing in this and several other regions over recent years [9, 10]. Infection is managed by the use of preventives in the macrocyclic lactone drug class, which, when administered regularly, kill larval worms before their development into adults.

Other arthropods affecting dogs (e.g., fleas and ticks) may be routinely managed with ectoparasiticides, which effectively prevent infestation and the transmission of many of the pathogens that they vector. One such compound is fluralaner, a member of the isoxazoline class, which acts as an antagonist of γ-aminobutyric acid receptors and glutamate-gated chloride channels, inducing neurotoxicity that results in paralysis and death of the affected arthropods. This toxicity is significantly more selective for arthropod than mammalian neurons [11, 12]. Fluralaner has also been shown to exhibit toxicity against mosquitoes, with lethal effects on the larval and adult stages of *Ae. aegypti* demonstrated via multiple routes [13]. More recently, the target of fluralaner in *Ae. aegypti* was identified as the resistance to dieldrin subunit of the ionotropic γ-aminobutyric acid receptor [14]. These lethal effects of fluralaner against *Ae. aegypti* indicate its potential efficacy as a novel compound for the interruption and/or prevention of transmission of mosquito-borne pathogens.

In dogs, fluralaner has a long elimination half-life and residence time in plasma, which enables 12 weeks of persistent efficacy against fleas and ticks after treatment with a single oral dose [15–17]. This efficacy relies on the attachment of the arthropod to the host and its ingestion of the active compound from the host’s circulatory system. Exposure to fluralaner rapidly kills ticks after attachment, which affects the transmission potential for *Borrelia burgdorferi* and *Babesia canis*, and may also reduce the transmission potential for *Anaplasma phagocytophilum*, *Ehrlichia canis*, and *Babesi microti* [18–21]. The efficacy of fluralaner against *Lutzomyia longipalpis*, the main vector of *Leishmania infantum*, has also been demonstrated through vector mortality [22]. For heartworm, as the duration of the blood meal of the mosquito vector is relatively brief, at least two feeding events are required for the parasite to complete its life cycle: one for the uptake of microfilariae by the mosquito vector from the peripheral blood of an infected host, and another at least 10 days later for the transmission of the infective third-stage larvae to a new host [23]. For compounds like fluralaner with a prolonged presence in the circulatory system of dogs, these feeding events offer opportunities to interrupt the heartworm life cycle when fluralaner is present in the host at concentrations lethal to the mosquito vector.

The aim of the present study was to evaluate the efficacy of fluralaner against *Ae. aegypti* blood-feeding, survival, and reproduction following the treatment of dogs with a single oral dose. To this end, mosquitoes were fed blood that was drawn from treated dogs or untreated controls once prior to treatment and weekly for 15 weeks thereafter.

## Methods

### Study design

The study protocol was approved by the Institutional Animal Care and Use Committee of the University of Georgia (protocol A2020 10-009). A randomized block design was utilized in this controlled efficacy study. Purpose-bred dogs 15 months of age were used. Mosquitoes were fed on blood drawn from each study dog (*n* = 12) prior to its assignment to any treatment group, and the percentage blood-feeding rate was determined for blood from each dog. None of the dogs had received any endectocide or heartworm prevention in the 60 days prior to the study. The dogs were sorted by mosquito blood-feeding rate and blocked into pairs. For each block, the treatment group was randomly assigned. No adverse events related to treatment were observed during the course of the study.

On study day 0, dogs in the treatment group (*n* = 6) were administered fluralaner in the form of Bravecto^®^ Chews (13.64% weight/weight fluralaner chewable tablets for dogs) in accordance with their weight and the label directions. As all the dogs fell within the same body weight range (4.9–7.1 kg), they were each administered 250 mg fluralaner. Dogs in the control group (*n* = 6) were handled similarly to the treated dogs, but received no fluralaner treatment. Immediately prior to treatment on day 0 and then weekly for the following 15 weeks (study weeks), a maximum volume of 25 ml blood was collected from each dog. Approximately 200–300 µl plasma was separated from this volume of blood and stored at − 80 °C for pharmacokinetic analysis, and 15 ml of the whole blood was used for the blood-feeding of mosquitoes. Blood for the feeding of mosquitoes was collected in heparin tubes, while that from which plasma was isolated for the pharmacokinetic analysis was collected in K2-EDTA-treated tubes.

### Mosquito blood-feeding

The blood collected as described in the previous section was used to feed adult *Ae. aegypti* mosquitoes (black-eyed Liverpool strain) [24] that had hatched 12 days earlier. The mosquitoes were maintained in an environmental chamber at 27 °C and a relative humidity of 75%, in accordance with standard procedures [25]. Water-jacketed membrane feeders were used for the feeding of adult mosquitoes on heparinized blood collected from the study animals. Adult mosquitoes were kept in three containers per study animal, each of which housed approximately 100 adult female mosquitoes. The adult mosquitoes were fed on 5 ml heparinized blood per container, and the membrane feeders were removed after 2 h. Mosquitoes in one of the three containers were used to assess blood-feeding rate, while those in the other two containers were maintained for a further 14 days to monitor survival and egg laying.

### Mosquito feeding rate

The mosquitoes were processed within 1 h of completion of blood-feeding to determine their feeding rate. One container per study animal was chilled at − 20 °C to immobilize the mosquitoes within, and each female mosquito was examined for visible engorgement of the midgut; the presence of any blood in the midgut was recorded as successful blood-feeding. Feeding rate was defined as the percentage of successfully blood-fed mosquitoes out of the total female mosquitoes per container (approximately 100).

### Mosquito survival

Two containers of adult, blood-fed mosquitoes per study animal were maintained for 14 days following blood feeding, a timeframe known to allow the development of infective third-stage *D. immitis* larvae from microfilaremia ingested during a blood meal under the conditions utilized in this study [26]. Mosquitoes were provided with cotton pads soaked with reverse-osmosis water and sugar cubes, which were replaced as needed. Dead mosquitoes were removed from each container daily for the first 4 days post-feeding and every other day thereafter. Dead mosquitoes were counted and sexed. On day 14 post-feeding, all of the remaining mosquitoes were counted and sexed to calculate a final survival rate. Adult mosquito survival was defined as the percentage of adult female mosquitoes surviving per container at each timepoint examined (1, 2, 3, 4, 6, 8, 10, 12, and 14 days post-blood-feeding) out of the total female mosquitoes per container (approximately 100).

### Mosquito egg laying

The adult mosquitoes in each container were provided with a 70-mm filter paper disc (Fisherbrand^™^ Qualitative P2; Fisher Scientific, Pittsburgh, PA) on which they could lay their eggs. The disc was pressed flush against the top screen of the container and kept damp with a pad of reverse-osmosis water-soaked cotton. The disc was removed and replaced with a fresh disc 3, 6, and 14 days following blood-feeding. Eggs were counted by an automated image capture and analysis process; Python script utilizing the OpenCV module and predefined parameters for the size and shape of *Ae. aegypti* eggs provided an estimated total number of eggs per disc. Egg laying per container was defined as the sum of egg counts for all three discs collected, and egg laying per mosquito was calculated from the number of live female adults remaining on the last day of collection.

### Plasma fluralaner concentration

Aliquots (100 µl) of dog plasma were extracted with acetonitrile containing a fixed concentration of D4-fluralaner (racemic). After shaking or mixing in a vortex for 10 min and centrifugation for 10 min, 400-µl aliquots of the supernatant were transferred into separate new 96-well plates and analyzed directly for* R*- and* S*-fluralaner. Fluralaner occurs as a racemic mixture of* S* and* R* enantiomers, the former being the active component of the drug and the latter the inactive one; analysis of both components is used to show the persistence of the compound in the circulatory system. The final extracts were analyzed by liquid chromatography–tandem mass spectrometry for* R*- and* S*-fluralaner using a triple quadrupole mass spectrometer in positive ion mode. For each analyte, any observed sample concentration below the limit of quantification (2.5 ng/ml) was reported as zero.

### Data analysis

Treatment efficacy was calculated using arithmetic means for blood-feeding rate and survival, and geometric means for egg laying, all with Abbot’s formula:

$$ {\text{Efficacy}}\,{\text{(\% )}}\,{ = }\,{100 } \times \,{{\left( {{\text{Mean}}_{{{\text{Control}}}} - {\text{Mean}}_{{{\text{treatment}}}} } \right)} \mathord{\left/ {\vphantom {{\left( {{\text{Mean}}_{{{\text{Control}}}} - {\text{Mean}}_{{{\text{treatment}}}} } \right)} {{\text{Mean}}_{{{\text{Control}}}} }}} \right. \kern-0pt} {{\text{Mean}}_{{{\text{Control}}}} }} $$

For all comparisons, significant differences between the values of control and fluralaner-treated groups were assessed using a linear mixed model with treatment group as a fixed effect. Comparisons of adult mosquito survival were made following blood-feeding for each study week, while all other comparisons were made for blood-feeding across all of the study weeks. Comparisons for mosquito egg laying were made using log-transformed egg counts. The two-sided level of significance was set at *P* ≤ 0.05 and the Holm-Šídák multiple comparisons test was selected as the post hoc test where significant differences were observed. All analyses were performed in Graphpad Prism 8.0 (GraphPad Software, La Jolla, CA).

## Results

### Mosquito feeding rate

**Fig. 1:**
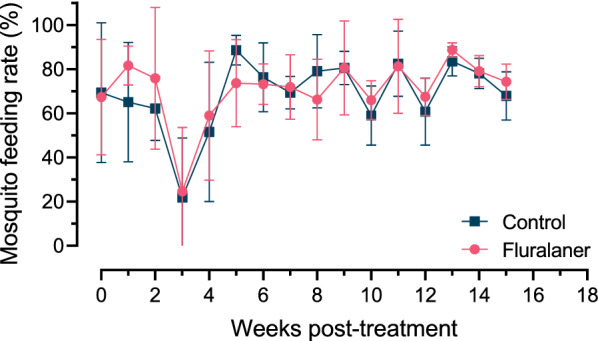
Feeding rate of adult female mosquitoes on blood collected on day 0 and then weekly from fluralaner-treated dogs (single oral dose) and untreated control dogs over a 15-week period. Data points represent the mean and SD for mosquitoes fed blood from six dogs per group (approximately 100 female mosquitoes per replicate). Data for study week 3 were excluded from analysis due to technical issues with the blood-feeding procedure. No significant differences were observed between the groups at any timepoint

No significant differences in mean blood-feeding rate were observed between mosquitoes fed on blood from control animals and fluralaner-treated animals (*P* > 0.05; Fig. 1; Additional file 1: Table S1). The rate of blood-feeding in each replicate ranged from 4.8 to 100% in the fluralaner treatment group and 3.1–98.8% in the control group, with a mean across all timepoints of 73.7% and 71.6%, respectively. Treatment efficacy against blood-feeding ranged from − 25.5 to 16.8%. An abnormally low rate of blood-feeding was observed for study week 3 (mean < 25%) due to a technical issue with the blood-feeding apparatus, and thus the data for this study week were excluded from analysis.

### Mosquito survival

**Fig. 2:**
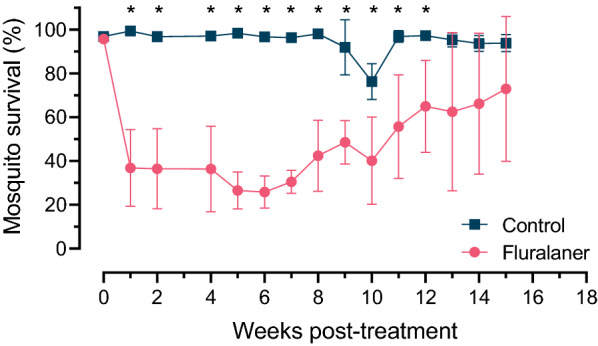
Rate of adult female mosquito survival 24 h post-feeding on blood collected on day 0 and then weekly from fluralaner-treated dogs (single oral dose) and untreated control dogs over a 15-week period. Data points represent the mean and SD for mosquitoes fed blood from six dogs per group (two replicates per dog; approximately 100 female mosquitoes per replicate). Asterisks indicate significant differences (*P* ≤ 0.05) between treatment groups at each timepoint

Feeding on blood from the fluralaner-treated dogs resulted in mortality of the adult mosquitoes, with significant differences in adult female mosquito survival between control and fluralaner treatment groups for each study week examined, at a minimum of one timepoint post-feeding (Additional file 2: Table S2). For the majority of study weeks, significant differences in mosquito survival were observed between control and fluralaner treatment groups at earlier timepoints post-feeding; there were fewer significant differences at later timepoints post-feeding because mortality was then similar between the control and the treatment groups. For blood collected for the first 12 weeks post-treatment of the dogs, rapid lethal effects of fluralaner were evidenced by a significant reduction in mosquito survival at 24 h post-feeding, with an efficacy range of 33.2–73.3% (*P* ≤ 0.041; Fig. 2; Additional file 2: Table S2; Additional file 5: Fig. S1). No differences in the survival of mosquitoes were observed when they fed on blood taken prior to treatment of the dogs with fluralaner (week 0; *p* > 0.05). Treatment efficacy 14 days after blood-feeding ranged from 14.2 to 91.4%, with significant differences observed for study weeks 1, 2, 5, 11, 12, 13, 14, and 15 (*P* ≤ 0.04).

### Mosquito egg laying

**Fig. 3:**
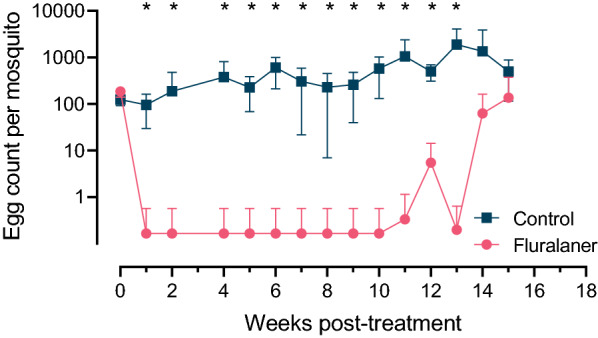
Number of eggs laid per mosquito after feeding on blood collected on day 0 and then weekly from fluralaner-treated dogs and untreated control dogs over a 15-week period; six dogs were treated with a single oral dose of fluralaner and six dogs comprised the control (two replicates per dog). Data points represent the geometric mean and SD calculated from the estimated number of mosquito eggs laid. Asterisks indicate significant differences between treatment groups at each timepoint (*P* ≤ 0.05)

A significant reduction in mean number of eggs laid by mosquitoes was observed for the fluralaner treatment group at timepoints up to and including 13 weeks post-treatment (*P* ≤ 0.0016; Fig. 3; Additional file 3: Table S3). Treatment efficacy remained above 99.8% during this period, but dropped to 95% by study week 15 (Additional file 5: Fig. S1). Egg counts in the fluralaner treatment group never exceeded 762 per mosquito for any replicate for any individual study week, while control egg counts ranged from three to 2814 per mosquito. No differences in egg production between the mosquitoes were observed when they fed on blood collected from the dogs prior to treatment (*P* > 0.05).

### Fluralaner plasma concentration

**Fig. 4:**
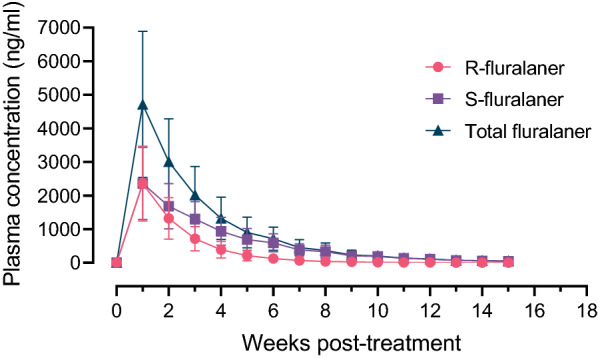
Plasma fluralaner concentration in dogs over a 15-week period following a single oral administration. Data points represent the mean and SD of the* R* and* S *enantiomers and total fluralaner concentration of six dogs

Fluralaner was detected in the plasma of all treated dogs at all timepoints during the 15-week study. The mean peak concentration of 4720 ng/ml, observed in study week 1, decreased over the course of the study to 47.7 ng/ml at the final timepoint (Fig. 4; Additional file 4: Table S4). The* R* enantiomer (inactive enantiomer) of fluralaner became undetectable in two of the treated dogs at week 10, and was undetectable in five out of the six treated dogs at week 15. The elimination half-life of total fluralaner was calculated to be 14.9 days. No fluralaner was detected in any of the untreated dogs at any timepoint, nor was fluralaner detected in the plasma of any dog prior to treatment.

## Discussion

The results of this study demonstrated effects of orally administered fluralaner (Bravecto^®^) in mosquitoes fed blood from treated dogs. The survival and fecundity of *Ae. aegypti* mosquitoes were reduced when they fed on blood collected weekly over a period of 12 weeks from dogs administered a single dose of fluralaner. During this period, a significant reduction in adult female mosquito survival was observed at 24 h post-blood-feeding, while egg laying was almost completely suppressed. These findings demonstrate a novel secondary effect of the ectoparasiticide fluralaner, which is currently labeled for use against fleas and ticks.

Concentrations of fluralaner that were capable of significantly reducing mosquito survival persisted for approximately six elimination half-lives in the circulatory system of the treated dogs. The relative lethal concentration 50% (LC50) of fluralaner at 24 h post-feeding was determined to be 58 ng/ml, which is in agreement with a mean LC50 of 32–100 ng/ml reported from blood-feeding studies conducted using *Anopheles* spp. [27]. A far greater LC50 for fluralaner at 24-h post-feeding (LC50 = 49 mg/ml) was reported for *Ae. aegypti* [13], but a sucrose solution was utilized in these feeding assays rather than blood, and the LC50 of the surface contact assays (LC50 = 13 ng/ml) agrees better with the result of the present study. Apart from the noted exception, the lethal effects of fluralaner in the circulatory system of the treated dogs appear to corroborate findings from in vitro systems.

A possible consequence of the lethal effect of fluralaner on blood-fed mosquitoes is that dogs receiving this treatment will serve as poorer reservoirs for heartworm transmission. Furthermore, the same formulation of fluralaner that remains effective against fleas and ticks for 12 weeks [15–17] conferred a significant mosquito-killing effect over the same timeframe in the present study. Thus, regular administration of fluralaner is likely to maintain this efficacy for as long as treatment with this ectoparasiticide is continued. It should be noted that, ideally, in an assessment of heartworm transmission potential the survival of adult female mosquitoes should be evaluated up to a plausibly infectious timepoint (i.e., 14 days after blood-feeding under the environmental conditions used in this study), at which point third-stage larvae, which are infectious to the mammalian host, are present in the mouthparts. While we monitored mosquito survival up to this timepoint, waning survival in the control groups over the 14-day period precluded meaningful comparisons. However, the rapid death of mosquitoes at earlier timepoints after their blood meal strongly indicated that the significant reduction in their survival was specifically related to their uptake of fluralaner.

Fluralaner does not, conversely, appear likely to prevent heartworm transmission to treated dogs, as treatment did not influence the rate of successful blood-feeding in mosquitoes at any point during the study. As third-stage heartworm larvae emerge from the mosquito mouthparts and rapidly enter the host during the infection process [28, 29], unlike pathogens vectored by ticks, which often require more than 24 h for transmission [18], an extremely rapid killing or deterrent effect due to fluralaner would be required to prevent the heartworm infection process.

Even more pronounced than the effect of fluralaner on the survival of blood-fed adults was its effect on their reproduction. Egg laying was almost completely suppressed in mosquitoes fed on blood collected from the dogs for up to 13 weeks after they were treated with a single dose of fluralaner. This suggests that mosquitoes feeding on dogs regularly administered fluralaner as an ectoparasiticide (i.e., every 12 weeks) will largely fail to reproduce and maintain their local population size. Fecundity is a key determinant of mosquito population modeling [30], and a significant reduction in egg laying is expected to likewise reduce the vector equilibrium population on a local level. This, in turn, should translate into a potential reduction in not just heartworm transmission, but also the transmission of other mosquito-borne pathogens. Owing to the relatively short dispersal ranges of some of the most significant vectors of *D. immitis*, which may not exceed a few hundred meters [31–33], it is not unreasonable to expect some degree of localized reduction in the population size and transmission potential of mosquitoes provided that they are sufficiently exposed to fluralaner-treated animals.

Routine fluralaner treatment may also fill gaps where conventional heartworm preventives fall short. Animals may benefit from possible reduced heartworm transmission via the passive mosquito-killing effect conferred by fluralaner (i.e., through blood-feeding), and while this is unlikely to directly prevent heartworm infection in dogs not receiving preventives, it may reduce their role as reservoirs for transmission. Additionally, the macrocyclic lactone-resistant isolates of *D. immitis* that have been identified over the past decade highlight an ongoing need for alternative control strategies [34–36]. Again, while fluralaner may not prevent infection with heartworm, it may assist in reducing its transmission from dogs in which resistant isolates are suspected while treatment is administered. Because this supplemental therapy targets the vector and not the parasite, its effect is not expected to depend upon macrocyclic lactone susceptibility, nor is it expected to exert selective pressure for resistance. Thus, the conventional use of fluralaner as an ectoparasiticide can have beneficial secondary effects, i.e. control of the mosquito vectors of heartworm and its transmission.

## Conclusions

*Aedes aegypti* mosquitoes exhibited significantly reduced survival and almost completely suppressed egg laying compared to untreated controls when fed blood collected from dogs over a period of 12 weeks following their treatment with a single oral dose of fluralaner. While blood-feeding was not affected by the fluralaner treatment, the mosquito-killing effects of fluralaner may contribute directly to a reduction in heartworm transmission potential in dogs receiving this ectoparasiticide, which may thus serve as a useful supplement to conventional preventives and therapies. Additionally, the pronounced inhibition of mosquito egg laying may contribute indirectly to a reduction in heartworm transmission potential in dogs, through a reduction in the local vector population.

### Supplementary Information

**Additional file 1: Table S1.** Mean rate of mosquito blood-feeding, range, and percentage efficacy prior to and 15 weeks following a single oral administration of fluralaner to dogs.

**Additional file 2: Table S2.** Mean survival rate of adult female mosquitoes, range, and percentage efficacy prior to and 15 weeks following a single oral administration of fluralaner to dogs.

**Additional file 3: Table S3.** Mean number of mosquito eggs laid, range, and percentage efficacy prior to and 15 weeks following a single oral administration of fluralaner to dogs.

**Additional file 4: Table S4.** Mean and SD of plasma fluralaner enantiomer concentrations over 15 weeks after a single oral administration to dogs.

**Additional file 5: Fig. S1.** Percent efficacy of fluralaner treatment compared to untreated controls prior to and 15 weeks following a single oral administration of fluralaner to dogs. Asterisks indicate significant differences between treatment groups at each timepoint for both survival and egg laying (*P* ≤ 0.05). Dagger represents a significant difference only in egg laying (*P* ≤ 0.05).

## Data Availability

The data that support the findings of this study are available from the corresponding author upon reasonable request.
