# Supplementary figures and images for: Treatment of dogs with Bravecto® (fluralaner) reduces mosquito survival and fecundity
Source: Parasit Vectors. 2023 Apr 28;16:147. doi: 10.1186/s13071-023-05682-8 (PMC10142166; doi:10.1186/s13071-023-05682-8)

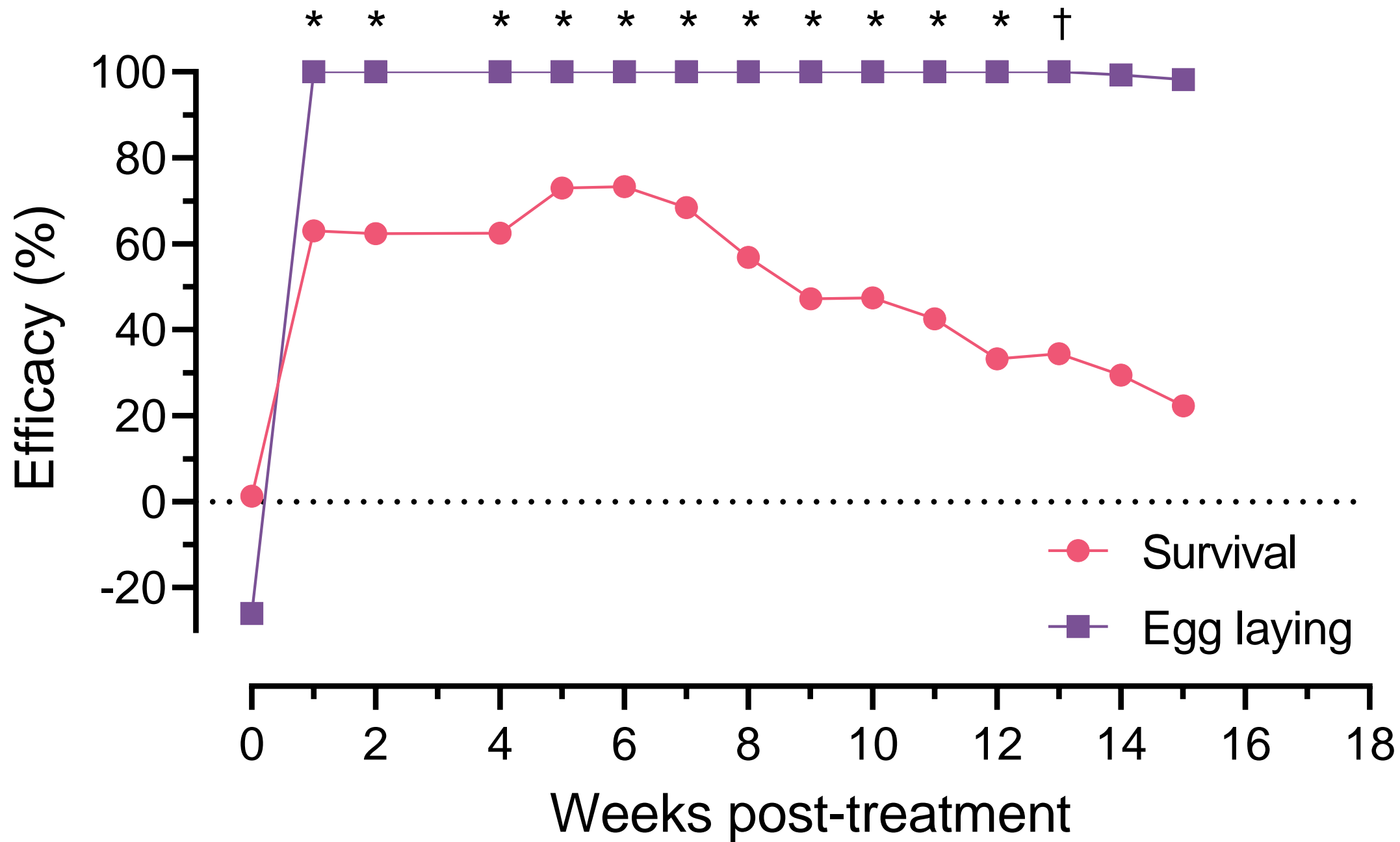

Supplement: Supplementary file 5 — Additional file 5: Fig. S1. Percent efficacy of fluralaner treatment compared to untreated controls prior to and 15 weeks following a single oral administration of fluralaner to dogs. Asterisks indicate significant differences between treatment groups at each timepoint for both survival and egg laying (P ≤ 0.05). Dagger represents a significant difference only in egg laying (P ≤ 0.05). [file 13071_2023_5682_MOESM5_ESM.pdf]
